# Changes in Socioeconomic Inequality of Low Birth Weight and Macrosomia in Shaanxi Province of Northwest China, 2010–2013: A Cross-sectional Study

**DOI:** 10.1097/MD.0000000000002471

**Published:** 2016-02-08

**Authors:** Leilei Pei, Yijun Kang, Yaling Zhao, Yue Cheng, Hong Yan

**Affiliations:** From the Department of Epidemiology and Health Statistics (LP, YK, YZ, HY), and Department of Nutrition and Food Safety Research (YC), School of Public Health, Xi’an Jiaotong University Health Science Center, Xi’an, Shaanxi, P.R. China.

## Abstract

Socioeconomic disparities in birth weights (BWs) are associated with lifelong differences in health and productivity. Understanding socioeconomic disparities in BW is presently of concern to develop public health interventions that promote a good start in life in Northwest China. In the study, our objective is to investigate the socioeconomic disparities in low and high BW from 2010 to 2013 in this region.

Those single live births were recruited using a stratified multistage sampling method in Shaanxi province from August to December 2013. Data were collected with a structured questionnaire and a review of birth certificates. Socioeconomic status (SES) was stratified based on the calculated household wealth index. Prevalence differences (PDs) and concentration indices (CIs) were used to depict the SES inequality of low BW (LBW) and macrosomia.

Information for 28722 single live births born were obtained in Shaanxi province. From 2010 to 2013, the overall rates of LBW decreased, and the difference in LBW across differing SES groups decreased by 0.7% (boys, 0.4%; girls, 0.8%). From 2010 to 2013, the overall rates of macrosomia increased by 14.3% (boys, 17.5%; girls, 7.8%), whereas the PDs in macrosomia across various SES groups remained unchanged. From 2010 to 2013, concentration indices for SES inequalities in LBW and macrosomia confirmed the results shown by differences in prevalence. Compared with mothers of high SES, those in low SES group were significantly older, less educated, engaged in farming with less availabile healthcare, and engaged in unhealthy lifestyles (eg, exposure to secondhand smoke) during pregnancy, regardless of the baby's sex.

From 2010 to 2013, in Shaanxi province, the negative association between socioeconomic status and LBW weakened. Rates of macrosomia were higher in those of high SES, but the SES disparities varied insignificantly over the same time. Our findings may provide valuable insights to direct healthcare policies for pregnant women to reduce inequalities in health, quality of life, and productivity for their children as they age into adulthood.

## INTRODUCTION

Birth weight (BW) is an important indication of mothers’ and neonates’ nutritional status, and may be the important determinant of infant's survival and future health, growth, and development.^1^ Studies have shown that both low BW (defined as weight <2500 g at the time of birth) and high BW (defined as weight >4000 g at the time of birth) are strongly associated with various short-term and long-term health problems, including delays in the development of motor, cognitive, and social skills, birth injuries, and obesity and chronic diseases as the child ages.^2–4^ It appears that conditions in utero and BW at birth may be a harbinger of much later problems in health, education, and achieving success in the labor market.^5,6^

It is commonly known that changes in physiological, nutritional, and sociocultural factors involved in the reproductive pattern of women are pivotal causes of neonatal BW variation.^7^ In fact, there is now fairly substantial evidences that BW tends to rise with socioeconomic status (SES) as reflected by income, education, and employment status.^6,8–9^ Given the long-lasting effects of early life conditions, it may be that BW, rather than the SES of the mother per se, more directly correlates with the socioeconomic disparity in health in adulthood. Thus, if the socioeconomic disparities in BW could be eliminated, longevity, quality of life, and labor market productivity might be improved.^6^

China is the populous country in the world, and economic development varies greatly by region, with particularly low SES levels in Northwest China. However, to our best knowledge, there are few, if any, investigations of BWs in Northwest China, or other areas of China, as they relate to the SES of different groups. With growing concern for promoting a good start in life for all its people, China's public health intervention programs presently seek to understand how SES disparities affect BW in Northwest China. According to the Sixth National Population Census of China of 2010, the population of Shaanxi province was ∼37 million, or around half that of all Northwest China.^10^ In 2013, a large population-based sampling survey was conducted in Shaanxi province to assess birth outcomes. In the current study, we investigated the SES discrepancies in low and high BWs from 2010 to 2013 in Shaanxi province of Northwest China. To some extent, careful measurement of such inequalities for low and high BW, one of the most salient manifestations of poor early life health conditions, may provide valuable policy insights.

## METHODS

### Study Design

A cross-sectional population-based epidemiological survey with the purpose to assess the birth outcomes, such as birth defects, BW, was conducted in Shaanxi province, in Northwest China, from August to December in 2013. Those infants born during 2010 to 2013 and their mothers were randomly sampled. Because of the population density of Shaanxi province, with rural and urban regions and a fertility rate of ∼9.73‰, a stratified multistage sampling method was adopted.

Within China's rural areas, there is a 3-level administrative structure consisting of county, township, and village. Independent of rural areas, in urban areas, the administrative structure consists of district, street, and community. For the present study, we first randomly selected from throughout the province 20 counties in rural areas and 10 districts in urban areas. In the rural areas, from each of the 20 counties, 6 villages each from 6 townships were then sampled randomly. Similarly, in the urban areas, from each of the 10 districts, 6 communities in each of 3 streets were selected randomly for sampling. A completely random sampling method was adopted to extract data for babies born between 2010 and 2013 and their mothers, specifically for 30 and 60 infants in each selected village and community, respectively.

It was expected that the sampling population would be ∼32,400 infants and their mothers. In our present study, the inclusion criterion was a single live birth. Excluded were cases of stillbirth, odinopoeia, abortion, multiple birth, or neonates without a recorded BW.

### Data Collection

All data collection was completed at the local village clinics and community health service centers. Ten field teams consisted of 10 to 12 trained investigators, a supervisor from Xi’an Jiaotong University Health Science Center, and a pediatric doctor from local maternal and child health hospital.

All maternal sociodemographic data, and information regarding access to healthcare, health status, and lifestyles during pregnancy were reported by the mothers of the sampled children through a precoded structured family questionnaire (Xi’an Jiaotong University Health Science Center). Only after obtaining written informed consent, face-to-face interviews were performed by the field investigators. Additional child-level data such as birth date and BW were found through a review of birth certificate. The supervisors reviewed each questionnaire immediately after completion for errors or missing values. The pediatric doctors of each team helped investigators collect information regarding birth outcomes. Our work was undertaken with the full support of the local hospitals and health administrative departments, as well as the Ministry of Health in Shaanxi province. The Human Research Ethics Committee of the Xi’an Jiaotong University Health Science Center reviewed and approved the study.

### Study Variables

The main outcome variables of study were low BW (LBW, neonates weighing <2500 g at birth, irrespective of gestational age), and macrosomia (>4000 g). The SES of the participants was assessed based on the Demographic and Health Survey household wealth index (HWI).^11^ Through principal component analysis, an HWI was calculated using 5 variables of family economic level: housing conditions, type of vehicle, income resources, and type and number of household appliances. The SES of the participants was ranked according to the value of the HWI, divided into thirds: low, medium, and high (poor, middle-income, rich, respectively).

#### Sociodemographic Characteristics

Neonatal characteristics such as sex, prematurity (yes or no), and gestation (weeks) were considered as the important explanatory (ie, independent) variables. Moreover, the following maternal sociodemographic variables were entered: maternal age at child-bearing (<25, 25–29, and ≥30 years); education (≤primary, secondary and ≥ high education), employment (farming and other occupations which included teacher, official, commercial and service staff, and professional), and residence during pregnancy. For residence, mothers who had been living in surveyed areas during pregnancy were recorded as permanent in the study, otherwise as transient.

#### Maternal Health Status in Pregnancy

During pregnancy, maternal health status is an important predictor of LBW and macrosomia. Thus, the mothers were required to report maternal health conditions such as anemia and negative (adverse) life events during pregnancy (yes or no, for both). Data regarding negative life events were obtained by asking the respondents whether they experienced bereavement, marriage crisis, family violence, unemployment, or debt disputes (using a yes/no response format) during pregnancy.

#### Maternal Lifestyles in Pregnancy

Data regarding maternal lifestyles during pregnancy, which included alcohol intake and passive (secondhand) exposure to smoke, were also acquired. In China, approximately two-thirds of men are regular smokers, whereas few women smoke; therefore, passive exposure to smoke by women is more pervasive.^12^ Passive exposure to smoke was defined as being exposed to another person's tobacco smoke for ≥15 min/d, >1 day/wk, and classified as yes or no.^13^ The mothers were also asked about the frequency (times/wk) and amount (grams/time) of a wide range of alcoholic beverages consumed during pregnancy, including liquor, wine, and beer. Alcohol intake during pregnancy was also considered a binary variable (yes or no).

#### Access to Healthcare in Pregnancy

We also collected the month antenatal care (ANC) was initiated, the number of ANC visits (<7 or ≥7), and folic acid supplementation before and during pregnancy, as a reflection of maternal access to healthcare.

### Statistical Analysis

First, we examined the baseline characteristics from 2010 to 2013 in Shaanxi province of Northwest China. Linear trend and intersubgroup differences in baseline characteristics were examined using *χ*^2^ and analysis of variance. Then, the prevalence differences (PDs) in LBW and macrosomia by sex and across SES were calculated to assess the inequality. To evaluate the changing trends of inequalities in LBW and macrosomia over time, after adjusting for all possible confounding factors (ie, sociodemographic characteristics, and maternal health status, lifestyles, and access to healthcare during pregnancy), the PDs across SES during 2010 to 2013 were obtained using a generalized linear model.

To provide a clear depiction of SES inequality of LBW and macrosomia, we further calculated overall and sex-specific concentration indices (CIs). A CI may range from −1 to 1, and is defined as twice the area between the concentration curve and the diagonal line of equality (the 45-degree line). The absolute value of the CI represents the severity of socioeconomic inequality, that is, the larger the absolute value, the greater the disparity. That CI equals zero means no socioeconomic inequality in LBW or macrosomia. CI <0 suggests that LBW or macrosomia is more concentrated in the low-SES group. At CI values >0, LBW or macrosomia is more concentrated in high-SES groups.

## RESULTS

### Baseline Characteristics

Of the total sampled population, 28,722 single live births occurring from 2010 to 2013 in Shaanxi province were eventually included in the study (Table 1), after the following exclusions: 2374 refused to participate; 349 were multiple deliveries; 756 resulted in loss of the fetus; and 199 had missing BW data. Of the live-born neonates, more than half were boys (54.8%) and premature births accounted for ∼5.9%. The average gestation was close to 39.6 weeks (range: 28–44 weeks). The mean ± SD of childbearing age of the mothers was about 28.0 ± 4.86, and more than 38.0% had at least a high school education. Among the mothers, 63.6% were involved in agriculture work, and 88.7% were members of the transient population.

**TABLE 1:**
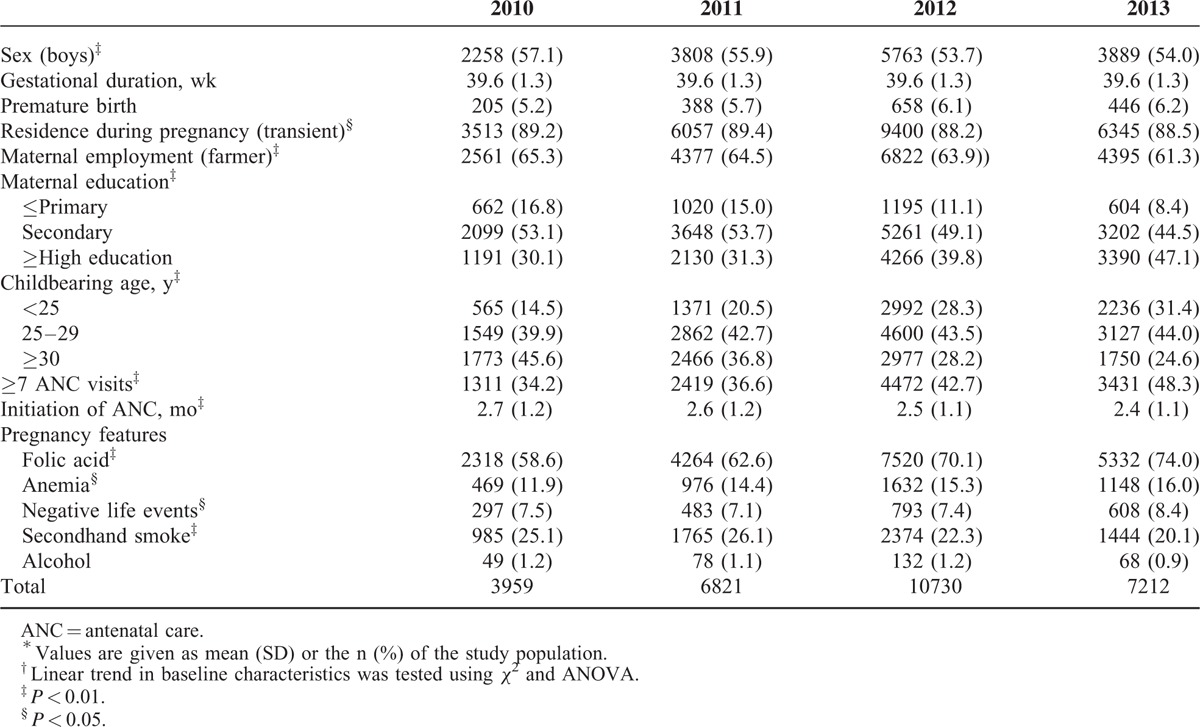
Baseline Characteristics of the Participants, Each Year From 2010 to 2013 in Shaanxi Province, Northwest China^∗,†^

With regard to access to healthcare during their pregnancy, ∼41.5% of the mothers attended ≥7 ANC visits, which were initiated an average of 2.54 ± 1.15 months after conception, and 67.7% of mothers had begun folic acid supplementation at least 3 months before the pregnancy. During pregnancy, 14.8% experienced anemia and 7.6% negative life events, ∼23.0% were exposed to secondhand smoke, and 1.1% drank alcohol. Based on HWI, the SES of the mothers was divided into three categories: low (∼32.8%), medium (32.9%), and high (33.3%).

### Socioeconomic Inequality in LBW and Macrosomia

#### Prevalence of LBW Across SES Groups by Sex Over Time

In the overall population, the prevalence of LBW decreased from 4.4% in 2010 to 3.6% in 2013. Among girls, the rates of LBW decreased from 4.6% in 2010 to 3.7% in 2013, a decrement rate of nearly 20.0%. Among boys, the prevalence of LBW reduced from 4.3% to 3.5% over the same time, a relatively lower descent rate of 16.3% compared with girls. Table 2 showed significant differences in the prevalence of LBW across SES groups by sex. Overall, differences in the prevalence of LBW among SES groups increasingly declined from 2010 to 2013. Disparities in the prevalence of LBW babies among SES groups in Table 2 were greater for boys than girls.

**TABLE 2:**
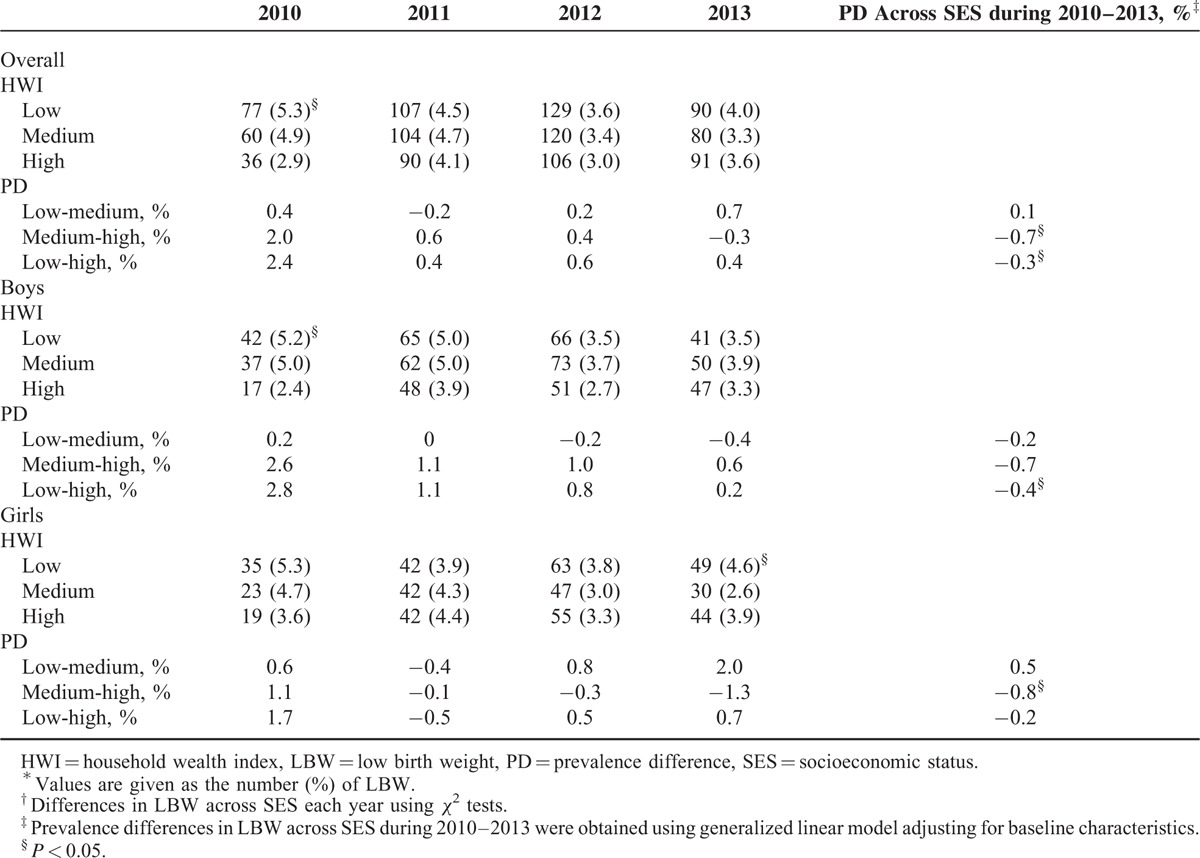
Prevalence of LBW Across SES Levels and by Sex, Each Year From 2010 to 2013^∗,†^

We used a generalized linear model, adjusting for confounding factors (sociodemographic characteristics and maternal health status, lifestyles, and access to healthcare during pregnancy), to determine the changes in PD in LBW across SES over the studied period. In almost all cases, the PD in LBW increasingly, compared with previous years, became weaker over time. From 2010 to 2013, the overall difference in LBW rates between the low and high SES groups decreased by ∼0.3%. Moreover, the difference in prevalence between the medium and high SES groups from 2010 to 2013 reduced by 0.7%. Among boys, we could observe a significant decrement in PDs between the low and high SES groups of about 0.4% from 2010 to 2013. Similarly, a reduction rate of ∼0.8% over the same time was observed in the PD between the medium and high SES groups among girls.

#### Prevalence of Macrosomia Across SES Groups by Sex Over Time

According to a pooled analysis, in the general population, the prevalence of neonatal macrosomia increased by 14.3% between 2010 and 2013 (boys, 17.5%; girls, 7.8%; Table 3). Table 3 displayed a nonsignificant SES discrepancy in macrosomia between different SES groups in almost all cases. After controlling for confounding factors, the generalized linear model also showed no changes in PDs in macrosomia across the SES groups from 2010 to 2013.

**TABLE 3:**
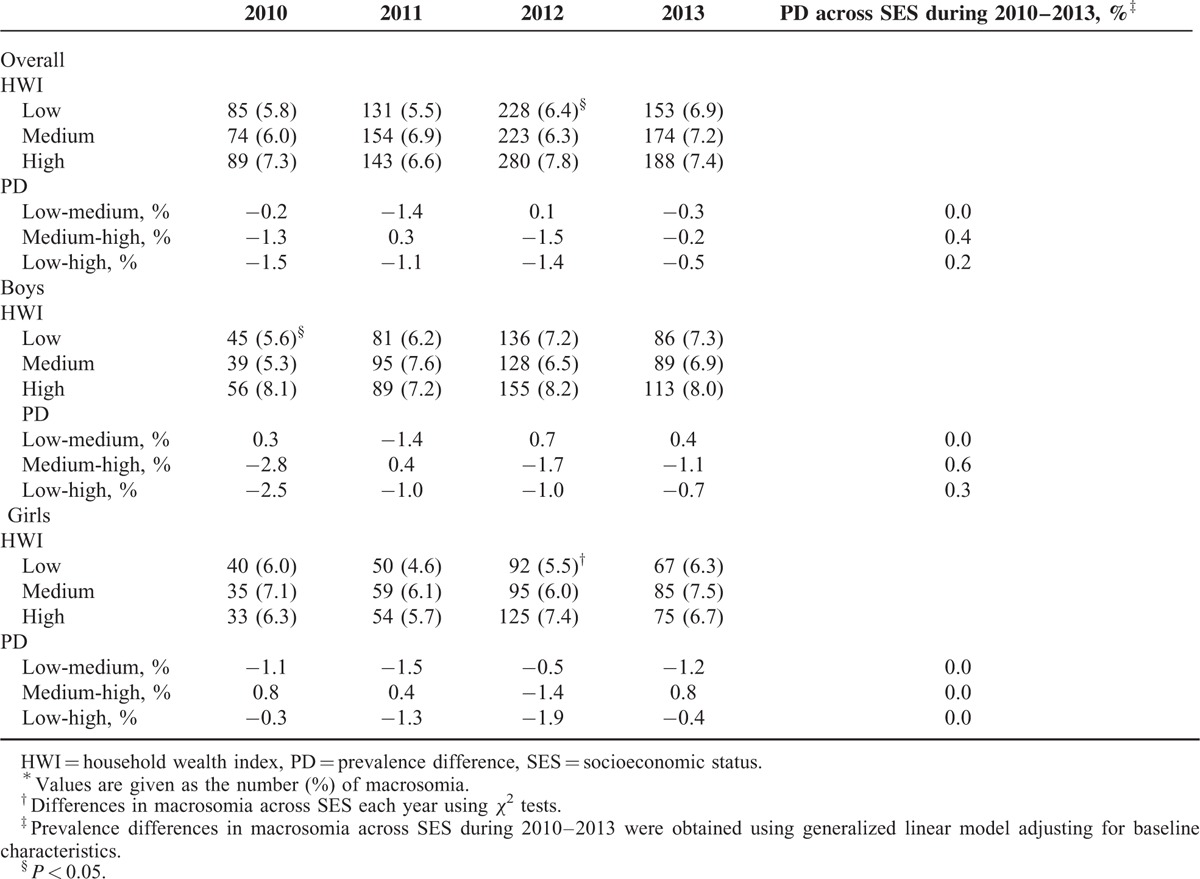
Prevalence of Macrosomia Across SES and by Sex, Each Year From 2010 to 2013^∗,†^

#### Concentration Index for the Whole Sample

**FIGURE 1:**
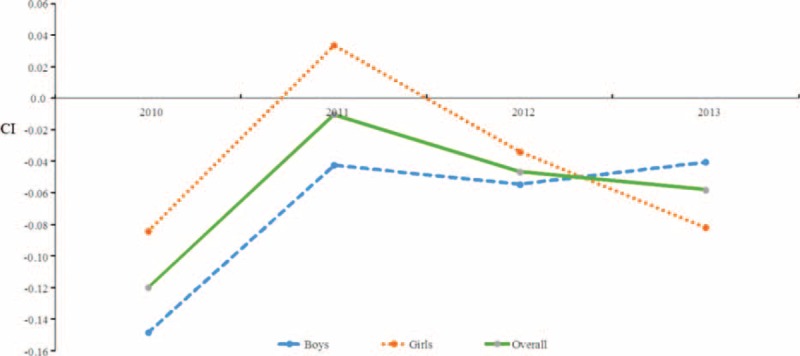
Trends in the concentration indices of low birth weight in the overall population and among boys and girls, from 2010 to 2013. CIs = concentration indices.

**FIGURE 2:**
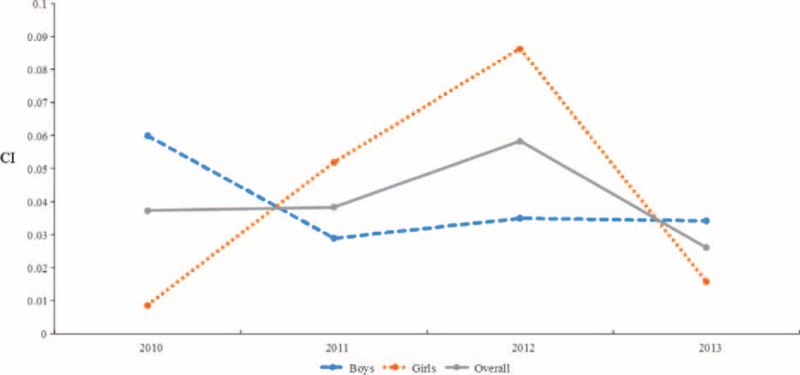
Trends in the concentration indices of macrosomia in the overall population and among boys and girls, from 2010 to 2013. CIs = concentration indices.

We also employed concentration indices to illustrate how SES might influence differences in LBW and macrosomia. As shown in Figures 1 and 2, CIs varied considerably by sex and over time. In the overall population, the CIs for LBW for each of the years 2010 to 2013 were, −0.120, −0.010, −0.047, and −0.058, respectively (Figure 1); the CIs for macrosomia were 0.037, 0.038, 0.058, and 0.026 (Figure 2). Regarding LBW for boys, each of the years from 2010 to 2013 showed CIs of −0.148, −0.043, −0.055, and −0.040, respectively; for girls, the analogous CIs were −0.084, 0.033,−0.034, and −0.082 (Figure 1). In reference to macrosomia, for boys, the CIs from 2010 to 2013 were 0.060, 0.029, 0.035, and 0.034, respectively; for girls, the CIs were 0.009, 0.052, 0.086, and 0.016 (Figure 2). Thus, the strength of the association between LBW and low SES appeared to weaken during this period, whereas there was no significant change in the association between macrosomia and high SES.

### Socioeconomic Disparities in Baseline Characteristics

We investigated the differences in sociodemographic characteristics and maternal health status, lifestyles, and access to healthcare during pregnancy across SES subgroups by sex (Table 4). Compared with mothers of high SES, those in low SES group were significantly older, less educated, engaged in farming with less available healthcare, and engaged in unhealthy lifestyles, regardless of baby's sex.

**TABLE 4:**
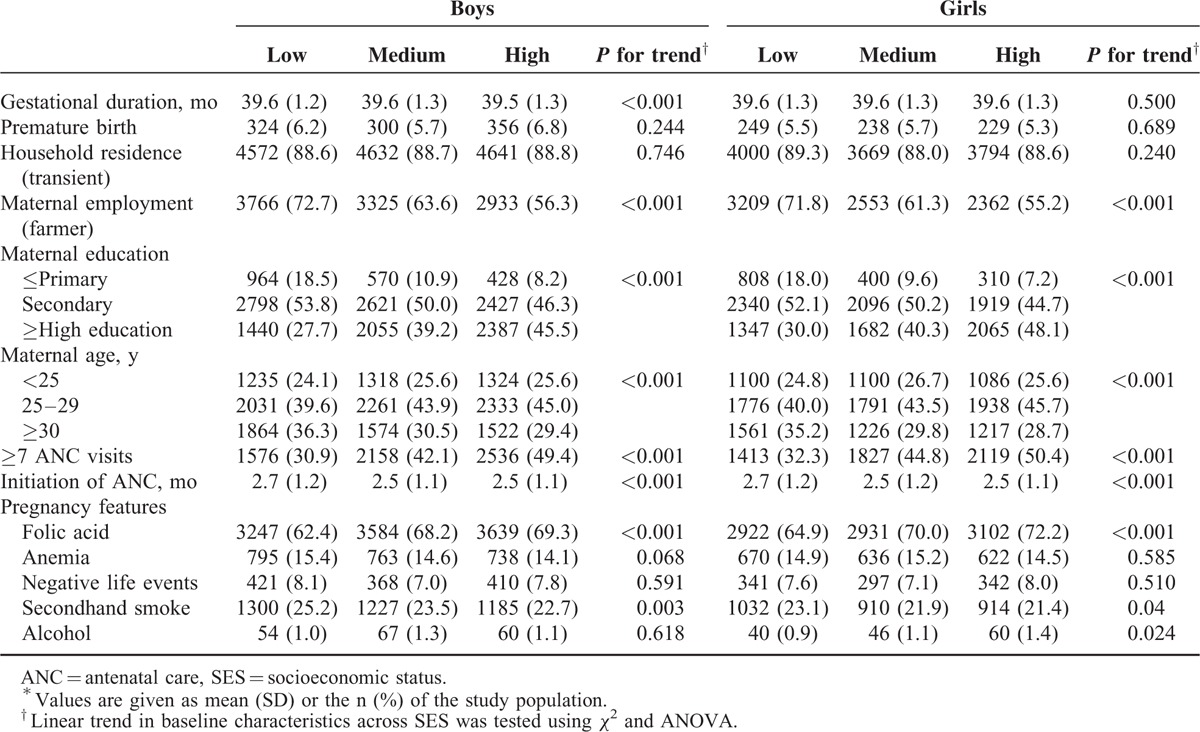
Characteristics of Mothers and Pregnancies Across SES Groups by Sex^∗^

Regarding the availability of healthcare, mothers of high SES were significantly more likely than low SES mothers to have taken folic acid and attend ≥7 ANC visits. In addition, there was a significant linear correlation between exposure to secondhand smoke during pregnancy and SES, and alcohol drinking and SES. There was no significant difference in maternal health status across SES among boys and girls.

## DISCUSSION

In the largest population-based study in Shaanxi province to date, we investigated whether socioeconomic disparities in LBW and macrosomia existed, and whether inequalities increased, decreased, or remained constant from 2010 to 2013. Our results revealed a clear link between LBW and SES, whereby mothers of low SES experienced greater rates of LBW babies than did mothers of high SES. However, this SES disparity in LBW decreased from 2010 to 2013. Furthermore, although macrosomia was far more likely in wealthier households, the trends in SES gradients remained sustained over time.

Our study confirmed that the overall prevalence of LBW had been reduced from 4.4% to 3.6% between 2010 and 2013. The figures in our study appear to be much lower than those of other studies from developing and developed countries in 2012, for example, 6.0% in Russia, 8.0% in Spain, 7.0% in Germany, and 10.0% in Japan.^14^ Furthermore, in the present study from 2010 to 2013, the rates of macrosomia rose from 6.3% to 7.2%, which was likely to be much higher than reported in Guangdong, China, in 2012 (2.3%) and South China in 2011 (5.6%).^15–16^ They were also higher than that reported by the 2008 World Health Organization global survey for India (0.5%), Vietnam (3.4), and Thailand (2.2%).^17^ Although LBW rates have been decreasing in Northwest China, macrosomia may become an important problem there.

To our best knowledge, this is the first documentation in China of a decreasing trend in SES disparity in LBW by sex, but unchanged SES gradient of macrosomia, using generalized linear model adjusted for risk factors including sociodemographic characteristics and maternal health status, lifestyles, and access to healthcare during pregnancy. These results were supported by the CI approach, in which results were consistent with those above. To date, there is substantial evidence from other countries that BW and SES are positively related; rates of LBW decrease as SES increases, and rates of macrosomia increase with measures of SES such as income, education, and employment status.^6,18^ For example, in Denmark, all measures of social disadvantage (including low education and poverty) were associated with decreased fetal BW.^19^ In studies conducted across larger geographic areas in the United States and Canada, where socioeconomic gradients were commonly considered to be low, similar socioeconomic disparities for BW had been reported.^20–21^ In general, our results seemed to be similar to previous research. Our study is unique in showing that, in Northwest China, SES disparities in LBWs have decreased annually, whereas the SES gradient of macrosomia has been sustained.

The bulk of epidemiological evidence suggested that changes in physiological, nutritional, and sociocultural factors involved in the reproductive pattern of women are the contributing factors of BW variation.^7,22–24^ To understand such complex SES-LBW and SES-macrosomia relationships in our study, we analyzed, for a 4-year period, the influence of sociodemographic features, and maternal health status, lifestyles, and access to healthcare during pregnancy across SES. According to the SES levels, firstly, we found that mothers of higher SES had achieved higher levels of education, were younger at childbearing, and were less likely to have an occupation in farming compared with women with poor SES. One study in Poland reported that being younger than 20 years was a protective factor for women against bearing LBW infants.^25^ Another study found that for mothers older than 40 years, there was a strong risk for extreme LBW.^15^ Highly educated mothers are more likely to have better jobs and higher incomes, as well as more knowledge of prenatal care. According to epidemiologic evidence, BW increased with mothers’ education level.^26–27^ Similarly, it was evident in our present study that from 2010 to 2013, maternal educational levels progressively improved, whereas the age at childbearing became lower and population performing agricultural work gradually decreased. These are the maternal sociodemographic characteristics that, at least in part, contribute to differences in BW related to SES and the trends we observed lead to higher BWs over time. ^26–27^

Second, it has been consistently shown that adequate prenatal care reduces the risk of prematurity and leads to increased BWs.^28,29^ The adequacy of prenatal care depends on establishing the mother as a patient early in the pregnancy, the number of prenatal visits, and the quality and content of care. In our present study, compared with the women of low SES, women from wealthier households attended prenatal care earlier and the percentage of women who had ≥7 prenatal care visits was higher. In addition, women of low SES were less likely to use folic acid as a supplement before and during pregnancy, which was found to be associated with BW.^30^ A significant temporal trend in improved utilization of ANC among mothers was observed in this study.

Third, socioeconomic-associated differences in BW are likely mediated by maternal lifestyles such as exposure to secondhand smoke and alcohol intake during pregnancy.^6,31–32^ Passive exposure to secondhand smoke is an important component in the association between SES and BW. This was shown by our present study, although this was found to decrease from 2010 to 2013. It is also worth noting that the percentage of women who drank alcohol during pregnancy was higher in women of the high SES group. However, previous studies have indicated that drinking alcohol in pregnancy might not be associated with BW growth.^33,34^ Unfortunately, it is not possible to draw conclusions from the present study regarding a linear trend in alcohol intake during pregnancy.

In general, mothers at lower socioeconomic levels are characterized by less education, inadequate care during pregnancy, higher unemployment and passive exposure to smoke, and other factors that may lead to reduced BW and other adverse birth outcomes. These findings support the view that economic growth also does not resolve differences in BWs. What is important is to eliminate the socioeconomic-associated differences in health by ensuring that those with lower SES have as much access to improved health, prenatal care, and healthier lifestyles before and during pregnancy as those of higher SES.

The primary strength of the present analysis is the large sample size (28722 single live births occurring from 2010 to 2013), which accounted for ∼9% of neonates in Shaanxi Province. Therefore, our results can be generalized to the entire province as well as Northwest China. Another strength of this study is that the BW data collected from birth certificates was precise to the nearest 10 g. However, this study of changes in BWs associated with SES is limited, in that data were analyzed for only the years 2010 to 2013, and a long-term study is warranted. Second, we lacked data on maternal prepregnancy body mass index, diet, and placental weight that could be important factors in a study of SES inequalities and BW. Nevertheless, the current study is the first and largest survey that has presently been conducted in Northwest China, and provides the best information on how SES affects LBW and macrosomia during this period in this geographical region.

In conclusion, although LBWs remained more prevalent in women of low SES compared with those of high SES, this negative association weakened from 2010 to 2013 in Shaanxi province of Northwest China. Rates of macrosomia were higher in those of high SES, but the SES disparities varied insignificantly over the same time. Disparities in BWs across SES levels may be more directly addressed by noting the differences in demographic, healthcare availability, and lifestyles during pregnancy that characterize these groups. Thus, efforts to improve SES-associated disparities in birth weight in Shaanxi province of Northwest China may require eliminating inequities in healthcare availability, encouraging healthy lifestyles, and providing health education for women before and during pregnancy.
